# Protocol of a randomized controlled trial on the effectiveness and cost-effectiveness of the PLACES intervention: a supported employment intervention aimed at enhancing work participation of unemployed and/or work-disabled cancer survivors

**DOI:** 10.1186/s13063-024-08441-x

**Published:** 2024-09-09

**Authors:** F. van Ommen, S. F. A. Duijts, P. Coenen, S. O. Dalton, A. Kliffen, R. van Hummel, A. G. E. M. de Boer, M. A. Greidanus

**Affiliations:** 1 Amsterdam UMC, Department of Public and Occupational Health, Location University of Amsterdam, Meibergdreef 9, 1105AZ Amsterdam, The Netherlands; 2 Amsterdam Public Health Research Institute, Societal Participation and Health, Amsterdam, the Netherlands; 3 Netherlands Comprehensive Cancer Organisation (IKNL), Department of Research & Development, Utrecht, the Netherlands; 4 Amsterdam UMC, Location Vrije Universiteit, Department of Medical Psychology, Amsterdam, the Netherlands; 5 Cancer Centre Amsterdam, Cancer Treatment and Quality of Life, Amsterdam, the Netherlands; 6 UMC, Location Vrije Universiteit, Department of Public and Occupational Health, Amsterdam, the Netherlands; 7 Amsterdam Movement Sciences Research Institute, Musculoskeletal Health, Amsterdam, the Netherlands; 8 Danish Cancer Institute, Cancer Survivorship, Copenhagen, Denmark; 9 Department for Clinical Oncology & Palliative Care, Danish Research Center for Equality in Cancer (COMPAS), Zealand University Hospital, Næstved, Denmark; 10 Dutch Social Security Institute, Haarlem, the Netherlands; 11 Cancer-Related Return-To-Work Consultancy and Guidance, Utrecht, The Netherlands

**Keywords:** Cancer survivors, Neoplasms, Employment, Unemployment, Return to work, Intervention, Randomized controlled trial, Clinical trial protocol, Work ability

## Abstract

**Background:**

Approximately onethird of cancer survivors encounter challenges reintegrating into the workforce, often experiencing involuntary unemployment and/or partial or full work disability following diagnosis and treatment. Returning to paid employment presents evident challenges due to uncertainties regarding work ability, perceived employer discrimination, and a lack of support, thereby risking social exclusion. However, interventions addressing return to paid employment among unemployed and/or work-disabled cancer survivors are scarce. Here, we describe the protocol of a randomized controlled trial (RCT), including a process and economic evaluation, evaluating the effectiveness and cost-effectiveness of the PLACES (unemPLoyed cAnCEr survivors Support) intervention aimed at supporting unemployed and/or work-disabled cancer survivors returning to paid employment.

**Methods:**

A two-armed RCT with a 12-month follow-up period will be conducted. Eligible participants: (1) are of working age (18–65 years), (2) are diagnosed with cancer between 6 months and 10 years ago, (3) are unemployed and/or partially or fully work-disabled, (4) have completed cancer treatment, and (5) are seeking paid employment and are motivated to initiate work immediately. Participants will primarily be identified through the Dutch Social Security Agency and the Netherlands Cancer Registry and recruited via healthcare professionals. Participants randomly allocated to the intervention group (*n* = 82) will receive the PLACES intervention: a tailored supported employment intervention based on the principles of Individual Placement and Support (IPS). This includes support in seeking, returning to, and maintaining paid employment. Participants allocated to the control group (*n* = 82) will receive care as usual. All participants will be asked to complete questionnaires, at baseline (T0), and after 3 (T1), 6 (T2), and 12 (T3) months of follow-up. The primary outcome is paid employment [yes/no]. Secondary outcomes are time until paid employment, change in working hours, work ability, quality of (working) life, and self-efficacy regarding return to work. Additionally, process and economic evaluations will be conducted.

**Discussion:**

We hypothesize that the PLACES intervention will be effective in obtaining paid employment, enhancing work ability, and improving quality of life. In addition, we expect the intervention to be cost-effective. If proven effective and cost-effective, actions should be taken to implement the intervention in usual care.

**Trial registration:**

NCT06028048.

**Supplementary Information:**

The online version contains supplementary material available at 10.1186/s13063-024-08441-x.

## Background

Nearly half of the 128,000 cancer diagnoses in the Netherlands in 2023 were among the working-age population (18–65 years) [1]. Advances in cancer screening, detection, and treatment have contributed to an increase in the number of cancer survivors [1–3]. Rising survival rates and an extended retirement age have led to and will contribute to substantial growth in the number of cancer survivors in the workforce. Both the diagnosis and treatment of cancer can substantially affect work participation of cancer survivors [4]. In several prospective studies, the risk of adverse work outcomes, such as unemployment and work disability, after cancer has been indicated. On average, 64% of cancer survivors return to work (RTW), indicating that about one third of survivors experience unemployment and/or partial or full work disability [5–10]. Notably, cancer survivors are 1.4 times more likely to experience unemployment than individuals without a history of cancer [8], and this specific increased risk persists even 10 years after diagnosis [11]. This not only has a profound personal impact on cancer survivors but also societal economic consequences (e.g., [4, 8, 11, 12]).

Many cancer survivors find themselves compelled to resume their work or re-enter the labor market due to financial concerns [13] (Greidanus et al. in preparation). Depending on country-specific legislation concerning work and sickness absence [14], individuals with an employment contract often have the opportunity to gradually increase their work tasks and working hours and develop new skills, which can boost their confidence and facilitate adaptation to their potentially changed circumstances [15]. In contrast, cancer survivors without an employment contract lack such opportunities and face additional challenges [15, 16]. That is, factors, such as uncertainty about their ability to work, lack of support from employers or colleagues, and perceived employer discrimination when applying for a new job, contribute to potentially more difficulties when re-entering the labor market (Greidanus et al. in preparation) [17]. Furthermore, socio-economically disadvantaged individuals, including those with lower education levels and income, tend to participate less in rehabilitation programs and report more unmet needs in the physical, emotional, work-related, and financial area [12]. Consequently, due to a cancer diagnosis, existing social inequalities may amplify, imposing a double burden on survivors who must navigate both health-related and work-related hardships after their cancer diagnosis and treatment [12].

Over the past decades, various interventions have been developed to support employed cancer survivors in returning to or maintaining their work [18]. However, interventions aimed at supporting unemployed cancer survivors are scarce, and there is limited evidence of their effectiveness [19]. As cancer survivors without paid employment experience distinct challenges [17], it is important to tailor interventions by addressing the specific needs of this particularly vulnerable group. In a systematic review of the limited number of interventions available for unemployed cancer survivors, it has been demonstrated that the effectiveness of these interventions is associated with the integration of job search, job placement, and workplace-focused components [19]. Supported employment is an example of an intervention that combines job search and placement assistance with workplace support, aiming to help unemployed individuals secure paid employment [20, 21]. A well-defined and studied form of supported employment is “Individual Placement and Support” (IPS) [21, 22], in which individuals are first placed in suitable employment, and then provided with workplace support. IPS has consistently proven to be effective for employment re-entry in various vulnerable patient groups, including those with severe mental illnesses and spinal cord injuries [23–25].

By combining IPS principles and already proven effective intervention components [19], we developed the PLACES (unemPLoyed cAnCEr survivors Support) intervention. In a randomized controlled trial (RCT), we will evaluate the following: (1) the PLACES intervention’s effectiveness on return to paid employment compared to care as usual (CAU) and (2) its cost-effectiveness. In this paper, we provide a comprehensive description of the PLACES intervention, and the description of an RCT protocol, including a process and economic evaluation.

## Methods

**Fig. 1:**
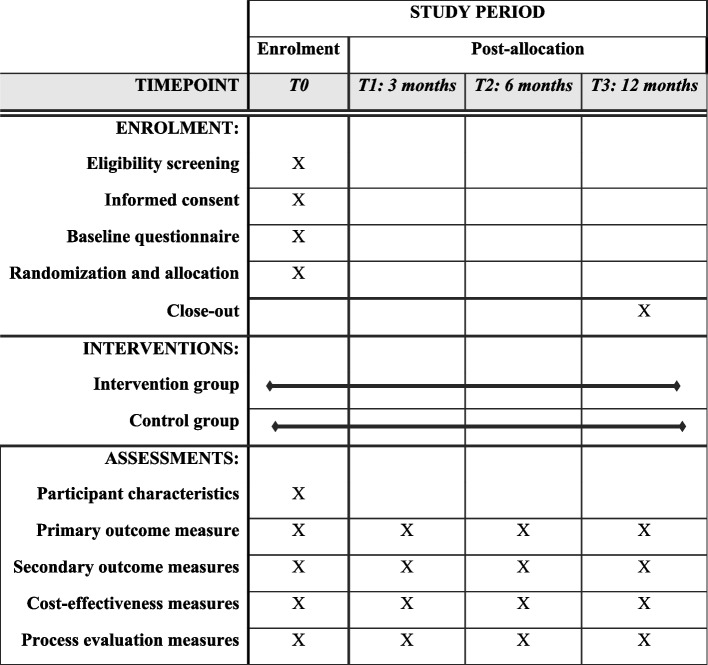
A schedule of enrolment, intervention allocation, and delivery of the intervention and assessments

**Fig. 2:**
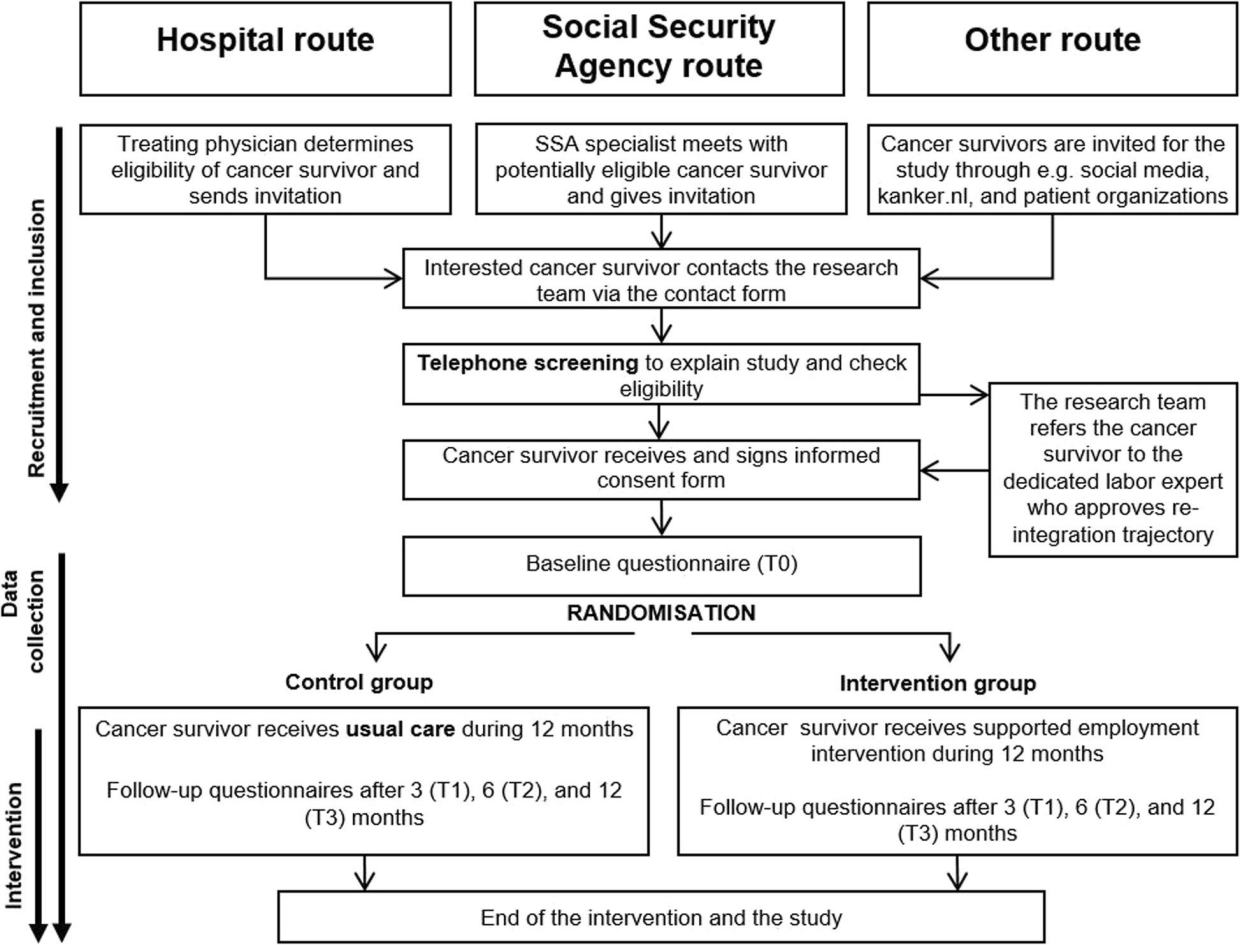
Overview of the study procedures

A two-armed RCT will be conducted, in which participants will be randomly allocated to (1) the PLACES intervention group or (2) the CAU control group. Participant recruitment, intervention delivery, and measurement timing are shown in Fig. 1. Participants will receive questionnaires at baseline (T0) and after 3 (T1), 6 (T2), and 12 months (T3) follow-up. The participant flowchart is shown in Fig. 2. The “Standard Protocol Items: Recommendations for Interventional Trials” (SPIRIT) 2013 statement was used to structure the design of this study [26] (Appendix S1). In addition to the trial registration (NCT06028048), all items of the World Health Organization Trial Registration Data Set are outlined in Appendix S2.

### Context

The study will be performed in the Netherlands, where the Social Security Agency (SSA) has two key responsibilities: (1) to determine the work ability of individuals and provide social benefits accordingly and (2) to facilitate reintegration trajectories to support individuals with re-entering the labor market after a period of absence. The type of social benefit an individual receives from the SSA is determined by their work ability, which is defined by their relative earning capacity. If a worker’s earning capacity is assessed as less than 35% of their previous salary, they are assigned a partial or full disability benefit. This applies to those who cannot continue their previous job or whose employment contract ends within the first 2 years of sick leave. The reintegration trajectories that the SSA facilitates are approved by labor experts based on the social benefit situation of individuals. The trajectories are provided on an individual basis, ensuring that they align with the specific needs and abilities of the individual.

### Participants and recruitment

Cancer survivors will be eligible to participate in this study if they:

1. Are of working age (18–65 years);
2. Were diagnosed with cancer between 6 months and 10 years ago;
3. Are currently unemployed and sick-listed and/or either partially or fully work-disabled;
4. Have completed primary cancer treatment (except long-term treatment such as hormone therapy);
5. Are currently seeking paid employment and motivated to initiate work immediately;
6. Are eligible for a reintegration trajectory at the SSA, based on their social benefit.

Cancer survivors who cannot speak, read, or understand Dutch and those who are diagnosed with basal cell carcinoma or a benign tumor will be excluded from participation.

Cancer survivors will be invited via three different routes (Fig. 2):

1. Hospital route: After identification via the Netherlands Cancer Registry (on items such as diagnosis, time since diagnosis and age), recruitment occurs by treating physicians in participating hospitals, who will provide an invitation package containing a flyer, information letter, contact form, and return envelope to cancer survivors who seem eligible based on information in their medical records (e.g., treatment and work-related information registered).
2. SSA route: Professionals from the SSA will provide the same invitation package to clients who disclose their cancer diagnosis to them (i.e., clients are not obligated to do so) and are motivated to initiate work immediately.
3. Other route: Digital invitations will be sent to cancer survivors through various channels including Kanker.nl (a Dutch online cancer platform), the Dutch Federation of Cancer Patient Organizations, and social media.

Invited cancer survivors have the option to respond by completing a digital (using a QR code) or hard copy (using a return envelope) contact form. They can express their interest in participating or provide a reason for nonparticipation. A researcher will contact interested cancer survivors by phone, providing further information about the study and screening for eligibility criteria 1 to 5. Eligible and willing cancer survivors will be referred to the SSA’s labor expert to determine eligibility for a reintegration trajectory (i.e., inclusion criterion 6). When the SSA confirms eligibility, the cancer survivor will receive an informed consent form via email or post (with return envelope), based on their preference. A model consent form is provided in Appendix S3.

### Sample size calculation

In a previous study of unemployed patients with spinal cord injury, participants who received IPS were significantly more likely to return to paid employment than those in the CAU group (30.8%; 95% CI 21.8–41.6 versus 10.5%; 95% CI 5.2–19.7, respectively) [25]. Based on these data, a power of 80% and an alpha of 0.05, a total of 148 cancer survivors are required to detect the same difference of 20% between the two groups (nQuery Advisor 7.0). Accounting for a 10% loss to follow-up, we aim to include 164 cancer survivors.

### Randomization

Participants will be randomly allocated to either the intervention or CAU control group (1:1), using Castor EDC [27], stratified according to region in the Netherlands and social benefit type. The allocation sequence will be generated by the executing researcher (FvO) in Castor EDC. The sequence is concealed for everyone until participants are allocated. Neither the participant nor the research team and those delivering the intervention will be blinded to the randomization.

### The PLACES intervention

#### Development

The PLACES intervention is a supported employment program, guided by trained and certified IPS coaches. The program was based on established principles of IPS (Table 1), which have proven effective in various other populations [23–25], with higher adherence to these principles leading to a more effective intervention [28]. Staying true to IPS principles was the foundation for the development of the PLACES intervention. Nevertheless, adjustments to three principles were necessary. Firstly, *zero exclusion* was not feasible due to funding structures within the SSA, and we recognized that cancer treatment is highly invasive, making it unrealistic for cancer survivors undergoing treatment to begin a new paid job (thereby excluding those still in treatment). To mitigate exclusion, we minimized the number of both inclusion and exclusion criteria. Additionally, *integration of rehabilitation and the healthcare system* is not yet possible. The PLACES intervention cannot be integrated into the oncological healthcare system since rehabilitation and oncological healthcare in the Netherlands are not yet adequately interconnected, unlike the more integrated approach seen in mental healthcare. Lastly, we could not guarantee *time-unlimited support* due to restrictions from the SSA and the duration of the study. The operationalization of the IPS principles for the PLACES intervention is shown in Table 1.

**Table 1:** Operationalization of the IPS principles for the PLACES intervention

Principle	Operationalization
Focus on competitive employment	The focus is on finding competitive employment in the community rather than volunteering or sheltered work for persons with disabilities
Eligibility based on client choice (zero exclusion)	Eligibility is based on the cancer survivor’s desire to work, rather than readiness or ability to work. Minimal exclusion is intended with exclusion criteria being: no social benefit from the SSA, unable to speak Dutch, or being diagnosed with basal cell carcinoma
Integration of rehabilitation and healthcare system	Re-turn, an organization specialized in reintegration after cancer, will train IPS coaches, equipping them with comprehensive knowledge on medical and social consequences of cancer diagnosis and treatment
Attention to worker preferences	Services will be based on each person’s preferences and choices, rather than the coaches’ or employer’s judgments. This means that preferences for work of the cancer survivor will guide the job search, rather than availability of existing jobs
Personalized benefits counseling	The IPS coach will help cancer survivors obtain personalized, understandable, and accurate information about their social security situation
Rapid job search	The cancer survivor will actively engage in job search and job development activities with their IPS coach, rather than conducting pre-vocational assessment or work readiness activities. The aim is to contact a potential employer within 30 days of the initial meeting
Systematic Job Development	Services will take place in a real-world setting. The IPS coach is expected to assist with finding a suitable workplace and providing the necessary “on the job” support
Time-unlimited and individualized support	Follow-along support will be provided by the IPS coach for 1 year, to support job maintenance

**Fig. 3:**
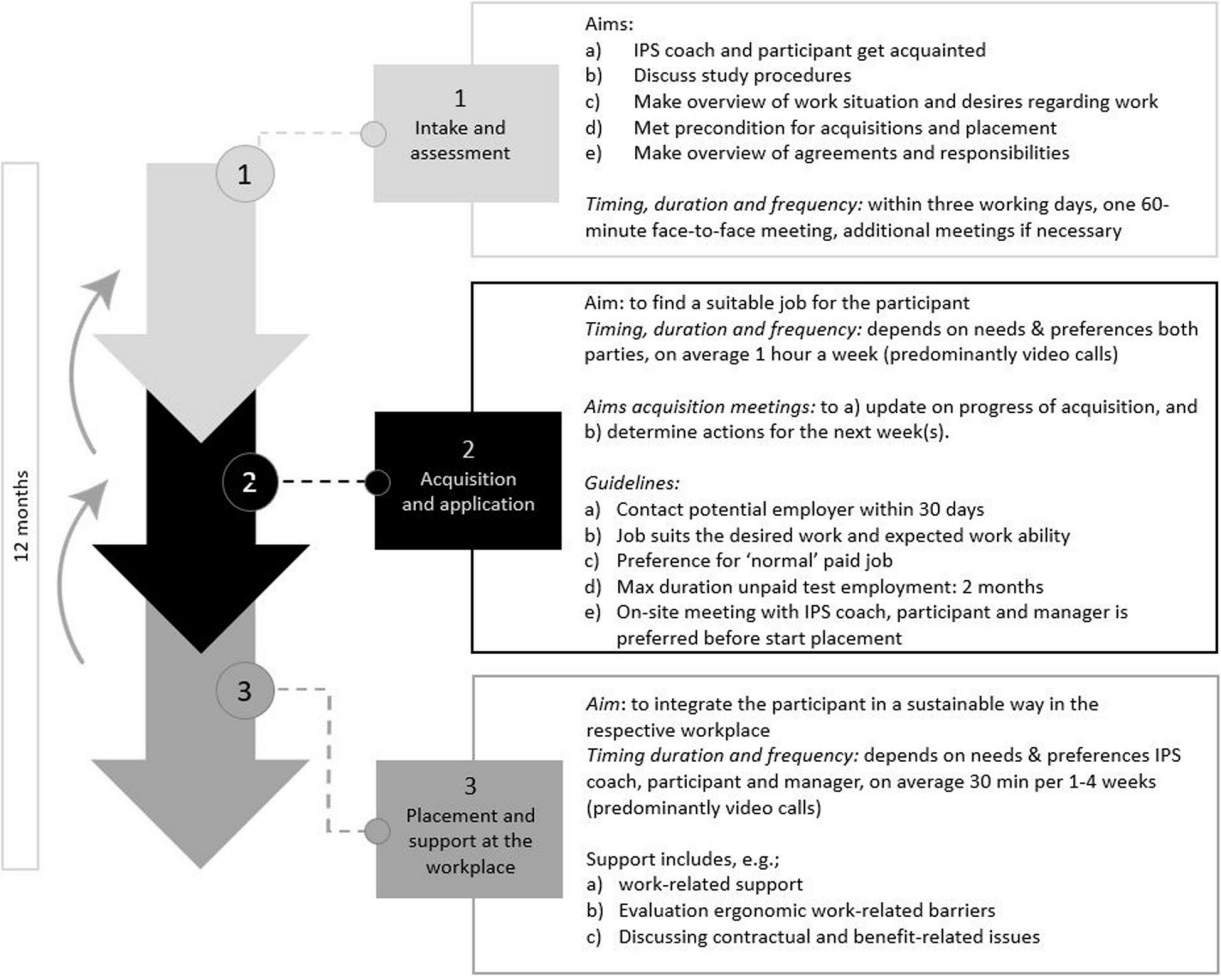
Phases of the PLACES supported employment intervention

In addition to these organizational adjustments of the IPS principles, the content of the PLACES intervention was tailored to the needs and preferences of unemployed and/or work-disabled cancer survivors. This was based on a systematic review [19] and focus group interviews (Greidanus et al. in preparation) with cancer survivors who had engaged in job-seeking activities for paid employment in the 2 years prior to the study (from a situation of unemployment or work disability). The review on interventions aimed at enhancing the work participation of cancer survivors [19], advocated, among others, for inclusion of the workplace even for individuals without an employment contract who are re-entering the labor market. This is in line with the principles of IPS on systematic job development and time-unlimited individualized support and resulted in the emphasis of support on the job and involvement of the employer during the intervention. The focus group interviews provided insight into facilitators and barriers encountered during the process of reintegration after cancer as well as the cancer survivors’ met and unmet needs throughout the reintegration process (Greidanus et al. in preparation). The insights gained from these interviews were used to formulate explicit recommendations for the coaches who perform the intervention, such as guidelines regarding the frequency and nature of the meetings. The specific guidelines for each phase of the intervention can be found in the content of the intervention and Fig. 3.

#### IPS coaches

Certified IPS coaches, with a background in higher vocational education (e.g., social work) and a completed training from the Dutch IPS knowledge center “Phrenos,” will deliver the intervention. Additionally, they will receive a PLACES-training and guidance from a commercial organization specialized in reintegration after cancer (i.e., Re-turn). The PLACES-training includes two videos with information on cancer survivors’ unique problems and needs, information on the organization of cancer care, and on study-specific information. After this training, all coaches have the opportunity to ask questions and discuss uncertainties regarding the intervention or the study with the executive researcher (FvO). Also, intervision meetings will be held twice a year with all coaches to collectively discuss cases and to learn from one another. We aim to recruit coaches from various Dutch regions to ensure national coverage. Each coach will operate within their own region and use their existing extensive network of employers to find suitable jobs for participants. All coaches will be supported and monitored by the reintegration organization.

#### Content of the intervention

Participants in the intervention group will be assigned to the reintegration organization that will conduct the PLACES intervention. The intervention comprises three consecutive phases with possible phase relapse (Fig. 3). The intervention will last a maximum of 12 months, starting after randomization, with each IPS coach having a maximum workload of 67 h per participant. This workload encompasses all tasks of the IPS coach, including administrative tasks, looking for a job and reporting to the SSA.

After a participant has been registered at the reintegration organization, an IPS coach will be assigned, and an initial meeting will be scheduled within three working days. In the “Intake and Assessment” phase (*phase 1*), the purpose is to become acquainted and discuss the participant’s work background and preferences. During this 60-min face-to-face meeting, the IPS coach and participant will also discuss their roles and responsibilities throughout the intervention. If necessary, additional meetings can be scheduled until the objectives of phase 1 are fulfilled.

In the “Acquisition and Application” phase (*phase 2*), the IPS coach guides the participant in finding a suitable job, including searching for vacancies, interacting with organizations, and coordinating the application process. The IPS coach may also assign tasks to the participant that can lead to finding a suitable job (e.g., writing application letters or creating or updating a LinkedIn profile). Meetings with the IPS coach will, on average, take place once a week via video calls to update each other on any progress and determine necessary actions. As a guideline, the IPS coach aims to contact a potential employer within 30 days of the initial meeting.

Once a suitable job has been found, the “Placement and Support at the Workplace” phase (*phase 3*) starts on the first day of work, during which the focus is on sustainable integration. The IPS coach will provide personalized support, adapted to the participant’s needs, which may include providing contractual advice and ergonomic support, and addressing work-related issues. The frequency of these meetings will depend on the participant’s needs. Optionally, if the participant prefers to do so, the employer or manager can join meetings and participate in the intervention.

### (Occupational) care as usual

Participants in the control group will receive CAU from the SSA, which may involve for example meetings with a reintegration expert or insurance physician. The aim of such meetings is usually to discuss or evaluate work ability and opportunities for RTW. The CAU from the SSA could also include the utilization of external reintegration services, such as job search assistance or vocational rehabilitation services. There are no restrictions for participants in the CAU control group to seek help from these or any other rehabilitation or medical services.

### Data collection

Data will be collected through the electronic data capture system Castor EDC [27], exported to and processed in SPSS 28.0, and stored at Amsterdam UMC secured servers. Participants will receive an email with a link to access the questionnaire. For those who prefer hardcopy questionnaires, paper versions will be sent to their preferred address, including a return envelope. Participants will be asked to complete the questionnaires within two weeks of receipt, with reminders per email after 1 week and per phone after 2 weeks. The executing researcher (FvO), principal investigator (AdB), and project leaders (MG and SD) will be given access to the final SPSS datasets. Here, each participant will be given an individual trial identification number. No adverse events, caused by our intervention, are anticipated. However, should any of such events occur, we will report them through the sponsor’s portal at the Amsterdam UMC. Also, serious adverse events will be documented and reported to the ethics committee.

## Study measures

An overview of all the measures and potential confounders, with detailed descriptions of the operationalization and the validated questionnaires, are presented in Table 2.

**Table 2:** Outcome measures and their assessment methods

Construct	# items	Description	Operationalization
**Participant characteristics**			
Age^a^	1	Participant’s age in years	Continuous
Gender^a,b^	1	Participant’s gender, with response options: male, female, or other	Categorical
Marital status	1	Marital status of participants categorized as: not married, married without children, married with children, living together with partner, living together with partner and children, living together with others, single with children, divorced, widow/widower, or other	Categorical
Children living at home	2	Living arrangement of participants (alone or together) and the number of children living at home	Continuous
Level of education^a^	1	The highest level of education completed by participants with eight answer options according to the Dutch education system, grouped into low, middle, and high, based on the International Standard Classification of Education (ISCED-97)	Categorical
Income^a^	1	Annual income of participants, with response options: < €30,000, €30,000–€60,000, €60,000–€100,000, and > €100,000	Categorical
Financial necessity to work	1	Whether it is a financial necessity for participants to work [yes/no]	Dichotomous
Time since diagnosis^a,b^	1	Time since cancer diagnosis at time of inclusion, calculated from the day (or month, if day is unknown) of diagnosis	Continuous
Type of cancer diagnosis^a,b^	1	The type of cancer diagnosed with answer options: breast cancer, skin cancer, colon cancer, lung cancer, prostate cancer, and other	Categorical
Type of cancer treatment^a,b^	5	Types of cancer treatment(s) received (e.g., surgery, radiation, chemotherapy, hormonal therapy, or a combination), duration of treatment, current treatment, and future treatment (e.g., hormone therapy)	Categorical
Importance of work^a^	1	Importance of work measured on a visual scale from 0 (not important at all) to 10 (very important)	Continuous
RTW expectation^a^	1	Participant’s estimation of the chances they will be at work in 6 months on a scale from 1 (no chance) to 10 (very high chance)	Continuous
**Primary outcome measure**			
Paid employment	1	Whether participant has engaged in paid employment since the last questionnaire [yes/no]	Dichotomous
**Secondary outcome measures**			
Time until paid employment^c^	1	Duration in calendar days between randomization and the first day of returning to paid employment	Continuous
Change in working hours^c^	1	Comparison between number of working hours at different time points (T1-T3)	Continuous
Health related quality of life	12	The Short Form Health survey (SF-12) is a widely used and validated measure of health-related quality of life (47)	The scale comprises 12 items covering physical health and mental health. Per dimension, the scores are added and transformed into a scale between – and 100. Higher scores indicate higher health related quality of life
Quality of working life	23	The Quality of Working Life Questionnaire for Cancer Survivors (QWLQ-CS) measures (1) meaning of work, (2) perception of the work situation, (3) atmosphere in the work environment, (4) understanding and recognition of the organization, and (5) problems due to the health situation [29]	The 23 items result in a standardized score of 0–100, with higher scores reflecting a better quality of working life
Work ability	1	The Work Ability Index (WAI) is a validated measure of work ability of which the first question will be used assessing participants’ estimation of their current work ability compared to their lifetime best on a scale from 0 to 10 (0 = cannot work at all and 10 = full work ability) [30]	Continuous
Self-efficacy regarding RTW	11	The self-efficacy regarding RTW scale assesses participants’ confidence in successfully RTW after health-related absence [31]. The scale is reliable with a Cronbach’s alpha between 0.90 and 0.96	11 items rated on a six-point scale from ‘totally disagree’ to ‘totally agree’. The RTW-SE score, with higher scores indicating more self-efficacy in RTW, is computed as the mean score of these items
Type of (paid) employment		Current work situation of participant (e.g., full time, part time, voluntarily unemployed, partly work-disabled)	Categorical
**Cost-effectiveness measures**			
Social benefits	1	Social benefit received by participant categorized as sickness benefit, partial or full work disability benefit	Categorical
Intervention costs		The costs of the reintegration trajectories at the SSA are equal to the sum of 67 h × hourly rate × number of participants. The cost of each individual trajectory is the same	Continuous
Productivity^c^	4	iMTA Productivity Costs Questionnaire (iPCQ) measures productivity costs related to health conditions, capturing absenteeism, presenteeism and performance at work (49)	Continuous
Occupational healthcare consumption	1	An Adapted iMTA Medical Costs Questionnaire (IMTQ) will assess self-reported occupational healthcare consumption categorized by different professionals (e.g., labor expert, reintegration services, physical therapist) and the number of appointment with each professional (50)	Continuous

^a^Used as potential confounder

^b^Used for subgroup analyses

^c^Only completed by participants with employment

### Participant characteristics

Sociodemographic and medical data, including age, gender, marital status, household and number of children, level of education, income, financial necessity to work, type of cancer, time since diagnosis, and cancer treatment, will be collected at T0. Some of these parameters will solely be used to describe the study sample, while others are used as potential confounder(s) or for subgroup analyses (see Table 2).

### Primary outcome measure

The primary outcome measure of this study is paid employment [yes/no] at any point during the 12-month follow-up. Participants will be asked at T1–T3 whether they have worked in paid employment since the previous questionnaire.

### Secondary outcome measures

Secondary outcome measures will include:

- Time until paid employment, defined as the number of days between randomization and starting paid employment. To calculate this, participants will be asked about the date they started their new job
- Change in working hours (if employed), measured by comparing the average numbers of hours worked weekly at different times of follow-up. For each work situation (e.g., part-time, voluntary), participants will be asked about the number of hours per week they work. The hours worked in paid employment will be used for the analyses
- Health-related quality of life, measured using the Short Form-12 (SF-12), which includes the subscales vitality, physical functioning, bodily pain, general health perceptions, and physical role functioning [32]. For the economic evaluation, the participants’ SF-12 health states will be converted to utility values, ranging from 0 (quality of life equal to death) to 1 (quality of life equal to optimal health), using the tariff of Brazier et al. [33]. Then, quality-adjusted life years (QALYs) will be estimated using the “area under the curve approach”
- Quality of working life, measured with the 23-item Cancer-specific Quality of Working Life Questionnaire (QWLQ-CS), which comprises five subscales: (1) meaning of work, (2) perception of the work situation, (3) atmosphere in the work environment, (4) understanding and recognition in the organization, and (5) problems due to the health situation [34]
- Work ability, assessed with a single question of the Work Ability Index (WAI), asking participants to estimate their current work ability compared with their lifetime best (0, cannot work at all; 10, work ability at its best) [35]
- Self-efficacy regarding RTW, measured using the 11-item self-efficacy scale [29]
- Type of paid employment, defined by the work situation. This will be reported with descriptive statistics. Answer options are as follows: fulltime, part-time, self-employed, studying, retired, involuntarily unemployed, voluntarily unemployed, partially or fully work-disabled, employed but on sick leave, unemployed and on sick leave, household/caring for others, and “other”

#### Costs

A cost-utility analysis (CUA) will be performed from an occupational healthcare perspective. The occupational healthcare perspective includes all costs associated with the formal Dutch occupational healthcare sector; i.e., intervention costs, occupational healthcare costs, absenteeism costs, and presenteeism costs. Intervention costs will be determined using a micro-costing approach. This means that detailed data will be collected about the number of resources used for developing, implementing, and delivering the intervention, which will in turn be valued using their respective unit prices. Occupational healthcare costs for all participants will be measured through self-reported questionnaires regarding the participant’s use of occupational healthcare services (e.g., insurance physician, labor expert), which will also be valued using prices of professional organizations. Absenteeism and presenteeism costs will be measured using the self-reported iMTA Productivity Costs Questionnaire (iPCQ) [36] and will be valued using gender-specific price weights [31]. The return on investment analysis (ROI) will be performed from the payer perspective. The costs for the payer perspective include the costs made by the SSA to provide the intervention, including the difference in costs for the intervention between the intervention and control group. Benefits will be defined as the mean difference in benefits costs. Information on the individuals’ benefits costs will be derived from the self-reported questionnaires and information about employment status.

#### Process evaluation

To assess the delivery and feasibility of the PLACES intervention, we will conduct a process evaluation using the model of Linnan and Steckler [37] (see Table 3). This model allows us to identify strengths and weaknesses of the intervention process that can be used to identify necessary improvements to enhance its effectiveness. Several key components of the intervention will be considered, including recruitment, reach, context, dose delivered, dose received, fidelity, and satisfaction.

**Table 3:** Overview of process measures according to the model of Linnan and Steckler

Components	Topic	Method of assessment
Recruitment	1. Recruitment route (i.e., hospital, SSA, other) 2. Type of social benefit at baseline (i.e., partially work-disabled, fully work-disabled or on sick leave at the end of their employment contract)	At T0, participants will report the recruitment route and social benefit type. Percentages per recruitment route and type of social benefits will be calculated for all participants and separately for the intervention and control group
Reach	1. The number of people invited, screened and who eventually participated 2. Reasons for non-participation or drop-out 3. Reach of IPS coaches 4. Reach of hospitals	The research logbook reports on: - Invitation, screening and non-participation numbers, with a distinction per labor region (participant reach) - Reasons for nonparticipation or drop-out at baseline, during screening, and throughout the 12-month follow-up period (participant reach) - The number of IPS coaches and hospitals contacted, along with reasons and numbers of nonparticipation (reach of IPS coaches and hospitals) Reach of IPS coaches will additionally be determined by the percentage of participating IPS coaches relative to the total IPS coaches in the Netherlands Reach of hospitals will additionally be determined by the percentage of hospitals relative to the total hospitals with an oncology department in the Netherlands
Context	1. Subgroup analyses to identify factors impacting the intervention implementation 2. Changes in the economic or political structures that influence the SSA 3.The influence of the labor market on intervention outcomes	Subgroup analyses are based on sociodemographic and clinical variables reported at T0 Economic or political changes that may influence the SSA will be reported on in the research logbook The influence of the labor market on the primary outcome will be assessed in interviews with the IPS coach
Dose delivered	Intervention content: 1. Meeting details 2. Total hours spend on delivering the intervention 3. Completion of all three phases 4. Completion of the PLACES & Re-turn training 5. Attendance of intervision meetings	In the IPS coach logbook coaches report on: - Phase completion (yes/no) - Number and duration of meetings - Type of meetings (e.g., face-to-face or video call) - Hours spent on delivering the intervention (e.g., meetings with participants, searching for a job, meeting with employers, on the job support) The research logbook will maintain information on the number of coaches that completed the training and attended the intervision sessions
Dose received	1. Job applications sent 2. Jobs started 3. Participant’s satisfaction with intervention the meetings: amount, duration, timing and content 4. The PLACES & Re-turn training	The IPS coach logbook reports on the number of applications sent and the number of jobs started by the participant The follow-up questionnaires (T1-T3) of participants in the intervention group will assess satisfaction with the intervention: - The satisfaction with the number, duration, and timing of the meetings with the IPS coach will be rated on a 5-point scale (Exactly right/not enough) - The content of the meetings will be rated as good/sufficient/bad Selected IPS coaches will talk about their experiences with the PLACES & Re-turn training. They will rate it as good, sufficient, or bad, and they will be asked if anything was missing from the training
Fidelity	1. Fidelity will be measured by assessing conformation to items and guidelines in the protocol, referred to as performance indicators: - IPS coach contacts participant within 3 working days - First intake meeting: within 2 weeks - First intake meeting: face-to-face - First intake meeting: 60-min duration - All aims of the intake phase are fulfilled - Clear agreements about responsibilities of participants and IPS coach in phase 2 - Contact with employer within 30 days after the initial meeting - Support by IPS coach in finding a suitable job - Support by IPS coach in preparing for job interviews - Support by IPS coach in preparing the start of the job - Meeting with employer, participant and IPS coach, if desirable 2. IPS coach perspective on application of IPS principles	The performance indicators will be assessed based on the IPS coach logbook. Additional information and elaboration on experiences will be drawn from interviews with IPS coaches The performance indicators will be scored as yes/not applicable (score 1) or no (score 0). All indicators will be weighted equally and converted into an overall value of performance. The intervention will be scored sufficient with a minimal score of 75% of the total In semi-structured interviews IPS coaches will be asked to what extent they think that they can apply the IPS principles to cancer survivors
Satisfaction	1. Participant’s satisfaction with the intervention 2. IPS coach’s satisfaction with the intervention 3. IPS coach’s perspectives on their role in supporting cancer survivors 4. Barriers and facilitators when providing the intervention	- Participant satisfaction scores of participants in the intervention group will be measured in the questionnaires at 3, 6, and 12 months on a scale from 1 to 10 - IPS coach’s satisfaction scores and their experiences will be assessed in interviews after the 12-month follow-up. They will be asked to rate their satisfaction on a scale from 1 to 10. They will be asked about their experience in their role in supporting cancer survivors and whether they felt confident in this role. They will also evaluate whether IPS is a suitable intervention for cancer survivors and what the barriers and facilitators are

Quantitative data will be collected using a questionnaire for participants, the IPS fidelity scale, an IPS coach logbook, and a research logbook (Table 3). The process evaluation questionnaire for participants includes questions about recruitment methods (T0) and assesses satisfaction with the intervention and the number, duration, and timing of the meetings (T1–T3). Performance indicators (e.g., completion of all three phases; see Table 3), established based on the IPS fidelity scale, will be evaluated using an IPS coach logbook. Each IPS coach will keep a logbook, providing comprehensive reports on various parameters such as meeting frequency and type per phase, as well as employment-related parameters, such as the number of job applications and interviews. Finally, a research logbook will be maintained, offering detailed information on several stakeholders of the study. Regarding participants, the number of people invited, screened, and included, along with the reasons for nonparticipation or drop-out will be reported. Additionally, the number of IPS coaches, hospitals, and SSA locations contacted for collaboration and the reasons and numbers of nonparticipation will be reported. Finally, qualitative data will be collected through semi-structured interviews with 4–6 IPS coaches and 4–6 participants, aiming to capture their experiences with the intervention and identify any barriers or facilitators influencing its implementation.

### Statistical analyses

To analyze nonparticipation data, we will tabulate the reasons for declining participation at baseline or during the follow-up period, aiming to gain a better understanding of the representativeness of the final study sample. Item frequencies and missing data for all items will be examined. Data cleaning will be performed to address inconsistencies as well as any missing values or improbable answers for the open-ended questions. The sociodemographic and clinical characteristics of the intervention and control group at baseline will be assessed and compared. Established scoring algorithms (as described in Table 2) will be used to calculate scores of the included scales. All analyses will be performed blinded. An independent researcher will code the intervention and control group as either 0 or 1 (with the person doing the analyses not knowing the key).

### Effect evaluation

To evaluate the effectiveness of the PLACES intervention compared to CAU at different time points, mixed-effect regression (either linear or logistic regression) with participant as a random intercept will be conducted. Since we stratified for “received social benefit” and the “labor region,” they will be included as covariates in the model. In all models, the main independent variable will be group allocation (i.e., intervention or control group), and both unadjusted and adjusted models will be presented. Additionally, the influence of time (T1, T2, T3) on the intervention’s effect will be examined (group*time interaction effect). The adjusted models will include potential confounders, such as age, gender, time since diagnosis, type of cancer, type of treatment, importance of work, RTW expectation [15, 38], and baseline value of the dependent variable.

In addition, subgroup analyses will be performed for gender (i.e., male, female or other) time since diagnosis (i.e., ≤ 3 years, > 3 years), type of cancer diagnosis (i.e., breast, colon or other), and type of treatment (i.e., locoregional therapy, systemic therapy or multimodal treatment). All analyses will be performed on an intention-to-treat basis. Per-protocol analyses will be carried out comparing participants in the intervention group who completed at least phase 1 of the intervention and had at least one meeting in phase 2 of the intervention, to participants in the control group.

We will use mixed-effects logistic regression to assess the primary outcome, paid employment [yes/no] at T1, T2, and T3. Odds ratios (ORs) with corresponding 95% confidence intervals (CIs) will be calculated to examine the effect of the intervention on the odds of paid employment.

Differences between the intervention and control group in secondary outcomes will be analyzed using linear or generalized linear regression analyses. Effect sizes and their 95% confidence intervals will be reported for all outcomes. To analyze the secondary outcome “time until paid employment,” a Kaplan–Meier survival analysis will be performed, and differences between intervention and control group will be tested with the log-rank test. In addition, the Cox proportional hazard model, or accelerated failure time analysis if applicable, will be applied to estimate hazard ratios and the corresponding 95% CIs, and the median time until paid employment for the intervention and control group will be determined if possible. All statistical analyses will be conducted using the IBM SPSS Statistics 28.0 [39] or R statistical software version 4.1.3 [40], and the statistical significance will be determined at a two-tailed significance level of 0.05.

### Cost effectiveness evaluation

The cost-utility analysis, in terms of QALYs, will be performed from the occupational healthcare perspective. Additionally, an ROI analysis will be conducted from the SSA perspective. For the cost-utility analysis, an incremental cost-effectiveness ratio (ICER) will be estimated by dividing the difference in occupational health costs between groups (∆C) by the corresponding difference in effects (∆E): (ICER = ∆C/∆E). To graphically illustrate the uncertainty surrounding the ICER, a cost-effectiveness plane (CE-plane) and a cost-effectiveness acceptability curve (CEAC) will be plotted. The ROI analysis will be conducted according to the recommendations of van Dongen and colleagues [41]. Various sensitivity analyses will be conducted to assess the robustness of the results (e.g., complete-case analysis).

## Discussion

In this article, the rationale and study protocol have been outlined regarding the effectiveness and cost-effectiveness of the PLACES intervention, a supported employment intervention, tailored for unemployed and/or partially or fully work-disabled cancer survivors. In our two-armed RCT, cancer survivors will be randomly assigned to the intervention group, receiving the PLACES intervention from specialized IPS coaches, or the control group, receiving CAU. Our hypothesis is that the supported employment intervention, compared to CAU, will be effective and cost-effective for enhancing return to paid employment and across various secondary outcome measures. Secondary outcomes include time until paid employment, changes in working hours, work ability, quality of life, quality of working life, and self-efficacy regarding RTW.

### Strengths and limitations

A strength of our study is that our outcome measures are in line with the recommended core outcome set for work participation [42]. According to this set, any type of employment (including self-employment) should be included as an outcome measure. Furthermore, according to this outcome set, intervention studies with participants who are absent from work due to a health problem, such as cancer, should include two outcomes: (1) “proportion of workers that return to work after being absent,” which is in line with our primary outcome measure of obtained paid employment, and (2) “time to RTW,” which is in line with our secondary outcome measure “time until paid employment.” Adhering to the core outcome set facilitates the comparability between studies on enhancing work participation of cancer survivors. The extensive process evaluation constitutes a second strength of the study. This allows us to draw valuable conclusions about the effectiveness of the PLACES intervention and to identify its key elements. It will help ensuring fidelity and addressing implementation barriers in the future. A final strength of the study is that, during the RCT, the intervention will be executed within the existing practical and financial structures of the SSA. This implies that no additional costs, compared to current SSA reintegration interventions, are anticipated. Moreover, if the intervention proves effective, its current embedding could facilitate potential future implementation [43].

The existing practical and financial structures of the SSA have, however, resulted in a limited applicability to all unemployed and/or work-disabled cancer survivors. For example, due to the SSA’s financial structures, we had to exclude some participants, such as formerly self-employed participants. This might influence the study’s external validity. Despite this limitation, conducting the intervention within the SSA framework is considered the most appropriate, as the SSA is the primary provider of re-integration trajectories for individuals seeking to re-enter paid employment in the Netherlands.

Another limitation of the study is that not all IPS principles can be fully met, as adaptations were necessary to accommodate the Dutch healthcare context. However, those criteria that could not be applied in the context of unemployed and/or work-disabled cancer survivors underwent minimal adaptations, guided by consultations with IPS experts from the knowledge center Phrenos, aiming to maintain fidelity to the IPS principles to the greatest extent possible.

### Impact of results

We believe that the intervention has the potential to enhance the work participation and quality of life of unemployed and/or work-disabled cancer survivors. The results of the RCT will contribute to the broader literature on the psychosocial needs and challenges faced by these cancer survivors. Additionally, the study will shed light on the effectiveness of supportive interventions tailored to this population, potentially addressing the need for more personalized interventions based on the characteristics of cancer survivors [18, 44–46].

Having paid employment has both personal and societal economic benefits. Based on existing evidence on supported employment interventions and effective intervention components to support unemployed and/or work-disabled cancer survivors in their RTW process, IPS seems to be a promising intervention for this population. The results from this RCT will improve the occupational healthcare of cancer survivors and contribute to the knowledge on the effectiveness of tailored interventions for the work participation of unemployed and/or work-disabled cancer survivors.

## Trial status

The current study protocol has been registered at ClinicalTrials.gov Protocol Registration and Results System (NCT06028048). The current manuscript describes the same protocol as registered in at ClinicalTrials.gov (version 1.0). The recruitment of participants started in September 2023 and is expected to be completed in December 2024. The results of the study are expected in 2026.

## Supplementary Information

Supplementary Material 1. SPIRIT Chls.

Supplementary Material 2. World Health Organization Trial Registration Data Set.

Supplementary Material 3. Consent Form.

## Data Availability

No data for this study are available yet. Data collected during this study will not be publicly available due to the privacy of the participants, who have not explicitly been informed about or approved data sharing. Data are available from the corresponding author upon reasonable request.

## Abbreviations

CAU: Care as usual
CE-plane: Cost-effectiveness plane
CEA: Cost-effectiveness analysis
CEAC: Cost-effectiveness acceptability curve
CI: Confidence interval
CUA: Cost-utility analysis
ICER: Incremental cost-effectiveness ratio
IMTQ: IMTA Medical Costs Questionnaire
iPCQ: IMTA Productivity Costs Questionnaire
IPS: Individual Placement and Support
NMB: Net monetary benefit
OR: Odds ratio
PLACES: UnemPLoyed cAnCEr survivors Support)
QALYs: Quality-adjusted life years
QLWLQ-CS: Cancer-specific Quality of Working Life Questionnaire
RCT: Randomized controlled trial
ROI: Return on investment
RTW: Return to work
SF-12: Short Form-12
SPIRIT: Standard Protocol Items: Recommendations for Interventional Trials
SSA: Social Security Agency
WAI: Work Ability Index
