# Supplementary material for: Protocol of a randomized controlled trial on the effectiveness and cost-effectiveness of the PLACES intervention: a supported employment intervention aimed at enhancing work participation of unemployed and/or work-disabled cancer survivors
Source: Trials. 2024 Sep 9;25:603. doi: 10.1186/s13063-024-08441-x (PMC11384714; doi:10.1186/s13063-024-08441-x)
Supplement: Supplementary file 2 — Supplementary Material 2. World Health Organization Trial Registration Data Set. [file 13063_2024_8441_MOESM2_ESM.docx]

**Appendix 3**. World Health Organization Trial Registration Data Set

**Table 3.** All items from the World Health Organization Trial Registration Data Set

| Data category | Information^32^ |
| --- | --- |
| Primary registry and trial identifying number | ClinicalTrials.gov NCT06028048 |
| Date of registration in primary registry | September 7, 2023 |
| Secondary identifying numbers | N/a |
| Source(s) of monetary or material support | This work is funded by the Alpe D’Huzes/Dutch Cancer Society |
| Primary sponsor | Academisch Medisch Centrum - Universiteit van Amsterdam (AMC-UvA) |
| Secondary sponsor(s) | N/a |
| Contact for public queries | Fenna van Ommen  Amsterdam UMC, 1105 AZ Amsterdam, The Netherlands  E-mail: f.vanommen1@amsterdamumc.nl |
| Contact for scientific queries | Fenna van Ommen  Amsterdam UMC, 1105 AZ Amsterdam, The Netherlands  E-mail: f.vanommen1@amsterdamumc.nl |
| Public title | Effectiveness of a supported employment intervention for unemployed and/or work-disabled cancer survivors. |
| Scientific title | Protocol of a Randomized Controlled Trial on the Effectiveness and Cost-effectiveness of the PLACES intervention: a Supported Employment Intervention Aimed at Enhancing Work Participation of Unemployed and/or Work-disabled Cancer Survivors |
| Countries of recruitment | The Netherlands |
| Health condition(s) or problem(s) studied | Unemployed and/or work-disabled cancer survivors |
| Intervention(s) | Intervention group: The PLACES intervention |
|  | Control group: Care as usual by the social security agency |
| Key inclusion and exclusion criteria | Cancer survivors will be eligible to participate in this study if they:   - are of working age (18-65 years); - were diagnosed with cancer between 6 months and 10 years ago; - are currently unemployed and sick-listed, and/or either partially or fully work-disabled; - have completed primary cancer treatment (except long-term treatment such as hormone therapy); - are currently seeking paid employment and motivated to initiate work immediately; - are eligible for a reintegration trajectory at the SSA, based on their social benefit. |
|  | Cancer survivors who cannot speak, read or understand Dutch and those who are diagnosed with basal cell carcinoma or a benign tumor will be excluded from participation. |
| Study type | Intervention |
|  | Allocation: randomized intervention model. Parallel assignment.  Masking: no masking (Open Label). |
|  | Primary purpose: Intervention |
|  | Phase II/III |
|  |  |
| Date of first enrolment | December 2023 |
| Target Sample size | 164 |
| Recruitment status | Recruiting |
| Primary outcome(s) | Paid employment (yes/no) [ Time Frame: Baseline (T0), 3 month (T1), 6 month (T2) and 12 month (T3) follow-up ]  The primary outcome measure is paid employment (yes/no) at any point during the 1-year-follow-up. Being in paid employment is operationalised as working in a paid job for at least one hour per week as defined by the central statistics office. |
| Key secondary outcome(s) | - Employment status [ Time Frame: Baseline (T0), after 3 months (T1), After 6 months (T2) and after 12 months (T3) ] - Time until paid employment [ Time Frame: Baseline (T0), after 3 months (T1), After 6 months (T2) and after 12 months (T3) ] - Change in working hours [ Time Frame: Baseline (T0), after 3 months (T1), After 6 months (T2) and after 12 months (T3) ] - Importance of work [ Time Frame: Baseline (T0), after 3 months (T1), After 6 months (T2) and after 12 months (T3) ] - RTW expectations [ Time Frame: Baseline (T0), after 3 months (T1), After 6 months (T2) and after 12 months (T3) ] - Work ability [ Time Frame: Baseline (T0), after 3 months (T1), After 6 months (T2) and after 12 months (T3) ] - Quality of life [ Time Frame: Baseline (T0), after 3 months (T1), After 6 months (T2) and after 12 months (T3) ] - Quality of working life [ Time Frame: Baseline (T0), after 3 months (T1), After 6 months (T2) and after 12 months (T3) ] - Self-efficacy regarding RTW [ Time Frame: Baseline (T0), after 3 months (T1), After 6 months (T2) and after 12 months (T3) ] |
