# Supplementary material for: Protocol of a randomized controlled trial on the effectiveness and cost-effectiveness of the PLACES intervention: a supported employment intervention aimed at enhancing work participation of unemployed and/or work-disabled cancer survivors
Source: Trials. 2024 Sep 9;25:603. doi: 10.1186/s13063-024-08441-x (PMC11384714; doi:10.1186/s13063-024-08441-x)
Supplement: Supplementary file 3 — Supplementary Material 3. Consent Form. [file 13063_2024_8441_MOESM3_ESM.docx]

**Consent Form**

For participation in the PLACES study

I hereby declare:

- to have been clearly informed about the nature, purpose, risks, and burden of the research, as well as about completing the questionnaires.
- to have had sufficient opportunity to ask questions about the research, and if applicable, that my questions have been satisfactorily answered.
- that I had enough time to decide to participate.

All information collected by the Amsterdam UMC, Department of Public & Occupational Health, will be treated confidentially. My research data will only be used for research purposes, kept for up to 15 years after the end of this study, and not disclosed to third parties. My data can only be accessed by the research team. Personal data cannot be traced back to me as an individual and will never be used in study documentation, reports, publications, or for insurance purposes.

I hereby voluntarily consent to participate in the PLACES study on the effectiveness of a program aimed at returning to paid work for people after cancer.

- I have the right to information about the study and the use of my research data.
- I am aware of the right to access data resulting from my participation.
- I have the right to be forgotten (meaning all data resulting from my participation will be destroyed by the researcher).

I can revoke this decision at any time, without having to provide a reason.

I want to participate in this study.

Name: ...............................................

Place: ...............................................

Date: ..../..../20...

Do you give the researchers permission to contact you again after this study, for up to 3 years after signing this form, for possible participation in follow-up research?

- Yes, I consent to this.
- No, I do not consent to this.

Please indicate below what applies (multiple answers possible):

- During the study, I would like to receive the PLACES project newsletter by email (maximum 2 times per year).
- After the study, I would like to receive the results of the research by email.
- I do not wish to receive the PLACES newsletter and the results of the research.
